# Quantitative MRI to Characterize the Nucleus Pulposus Morphological and Biomechanical Variation According to Sagittal Bending Load and Radial Fissure, an *ex vivo* Ovine Specimen Proof-of-Concept Study

**DOI:** 10.3389/fbioe.2021.676003

**Published:** 2021-06-09

**Authors:** Jean-Philippe Deneuville, Maksym Yushchenko, Tanguy Vendeuvre, Arnaud Germaneau, Maxime Billot, Manuel Roulaud, Mathieu Sarracanie, Najat Salameh, Philippe Rigoard

**Affiliations:** ^1^PRISMATICS Lab (Predictive Research in Spine/Neuromodulation Management and Thoracic Innovation/Cardiac Surgery), Poitiers University Hospital, Poitiers, France; ^2^Institut Pprime UPR 3346, CNRS – Université de Poitiers – ISAE-ENSMA, Poitiers, France; ^3^Department of Biomedical Engineering, Center for Adaptable MRI Technology (AMT Center), University of Basel, Allschwil, Switzerland; ^4^Department of Spine, Neuromodulation and Rehabilitation, Poitiers University Hospital, Poitiers, France

**Keywords:** intervertebral disc, nucleus biomechanics, quantitative MRI, low back pain, disc degeneration, radial fissure, mechanical diagnosis and therapy

## Abstract

**Background and context:** Low back pain is a dramatic burden worldwide. Discography studies have shown that 39% of chronic low back pain patients suffer from discogenic pain due to a radial fissure of intervertebral disc. This can have major implications in clinical therapeutic choices. The use of discography is restricted because of its invasiveness and interest in it remains low as it represents a static condition of the disc morphology. Magnetic Resonance Imaging (MRI) appears to be less invasive but does not describe the biomechanical dynamic behavior of the fissure.

**Purpose:** We aimed to seek a quantitative MRI protocol combined with *ex vivo* sagittal loading to analyze the morphological and biomechanical changes of the intervertebral disc structure and stress distribution.

**Study design:** Proof of concept.

**Methods:** We designed a proof-of-concept ovine study including 3 different 3.0 T-MRI sequences (T_2_-weighted, T_1_ and T_2_ mapping). We analyzed 3 different mechanical states (neutral, flexion and extension) on a fresh ovine spine specimen to characterize an intervertebral disc before and after puncturing the anterior part of the annulus fibrosus. We used a mark tracking method to calculate the bending angles and the axial displacements of the discal structures. In parallel, we created a finite element model to calculate the variation of the axial stress and the maximal intensity shear stress, extrapolated from our experimental boundary conditions.

**Results:** Thanks to an original combination of specific nuclear relaxation time quantifications (T_1_, T_2_) of the discal tissue, we characterized the nucleus movement/deformation into the fissure according to the synchronous mechanical load. This revealed a link between disc abnormality and spine segment range of motion capability. Our finite element model highlighted significant variations within the stress distribution between intact and damaged disc.

**Conclusion:** Quantitative MRI appears to provide a new opportunity to characterize intra-discal structural morphology, lesions and stress changes under the influence of mechanical load. This preliminary work could have substantial implications for non-invasive disc exploration and could help to validate novel therapies for disc treatment.

## Introduction

Low back pain is the leading cause of disability worldwide (Vos et al., 2016) and represents a dramatic economic burden for western countries (Maetzel and Li, 2002; Walker et al., 2003; Dagenais et al., 2008). Back pain is frequently associated with intervertebral disc degeneration, defined as “an aberrant, cell-mediated response to progressive structural failure” (Adams and Roughley, 2006). Several pathways can lead to disc degeneration (Adams and Dolan, 2012). One of them, starts from a centrifugal (from center to periphery) and radial fissure of the annulus which alters disc stress distribution (McNally et al., 1996) and creating a stress gradient between the posterior annulus and the nucleus pulposus (Stefanakis et al., 2014). These mechanical changes can modify the cellular activities leading to dysregulation of the TIMP/MMP expression (TIMP for Tissue Inhibitor of Metallo-Proteinase and MMP for Matrix Metallo-Proteinase) (Le Maitre et al., 2004, 2007). This, in turn, leads to the acceleration of the normal ageing nucleus dehydration (Antoniou et al., 1996). Such modifications can stimulate the nociceptors naturally present in the outer third of the annulus (García-Cosamalón et al., 2010) or those which proliferate alongside the fissure (Coppes et al., 1990, 1997; Lama et al., 2018). All of these phenomena define one structural substrate of discogenic backpain.

Aiming to reverse the pathological status of the disc, multiple treatment options are available, ranging from conservative management to interventional therapies. Robust concepts supporting physiotherapy and manual therapies base their intervention on the presence of a directional preference (McKenzie, 1981; McKenzie and May, 2003; Laslett et al., 2005), which implies a dynamic disc theory. From a clinical perspective, directional preference is a direction of movement alleviating patient pain, while the other directions have no effect or worsen the pain. When observed on a patient with back pain, this type of clinical sign is specific (94%) to discogenic pain (Laslett et al., 2005) and appears to function as an effective guide for treatment (May and Aina, 2012; May et al., 2018). In addition to disc surgery, which is limited to refractory patients, interventional therapies include chemonucleolysis using chymopapain (Javid et al., 1983) – historically the first intradiscal injected drug – collagenase, chondroitinase (Ishibashi et al., 2019), ozone (Paradiso and Alexandre, 2005), radiopaque gelled ethanol (Hashemi et al., 2020), and thermocoagulation (Freeman, 2006). Other novel intradiscal therapies, such as neurotrophic growth factor (Knezevic et al., 2017), platelet-rich plasma (Li et al., 2017) and stem cell (Meisel et al., 2019) appear promising but require further and large-scale validation. Treatment indication can be outlined by correlating a clinical phenotype to a specific morphological disc profile. However, this task becomes extremely challenging when a degenerative process occurs among pain-free and healthy subjects (Brinjikji et al., 2015).

As adequate treatment requires precise diagnostics and as the standard MRI fails, one has to fall back to discography. This procedure has historically been accepted as the reference standard imaging procedure for the diagnosis of discogenic pain. By injecting a contrast agent into the central nucleus, this procedure triggers a mechanical distension of the inner annulus, reproducing the concordant patient pain (Bogduk et al., 2013). By assessing the spreading of the contrast agent with an X-Ray or a CT-scan, the clinician can identify a centrifugal nucleus radial fissure. Discography studies have demonstrated that 39% of patients with chronic low back pain present discogenic pain associated with an annular radial fissure at the level of the injured disc (Manchikanti et al., 2018). The extent of the fissure can be graded using the Dallas classification (Sachs et al., 1987). Furthermore, patient pain has been shown to be proportional to the extent of the fissure toward the periphery (Vanharanta et al., 1987; Moneta et al., 1994). However, since discography is performed on patients in prone position, it enables the physician to analyze only a static aspect of the annular fissure. It provides no information about the potential mobility of the nucleus and the dynamic evolution of the fissure. Although this procedure has been modified to avoid any degenerative process (Bogduk, 2013; McCormick et al., 2019), some studies still show that discography could provide negative side-effects (Carragee et al., 2009). Because of these definitive disadvantages, its use has been restricted to a few highly specific conditions, and substantial efforts are being placed in the development of non-invasive markers (Aprill and Bogduk, 1992).

In recent years, Thompson et al. (2009) described the connection between positive discographies on more than 2400 discs and signal intensity alterations observed via Magnetic Resonance Imaging (MRI) on T_1_- and T_2_-weighted scans, corresponding to inflammation of the vertebral endplate, named “MODIC sign” (Modic et al., 1984). Such signs are associated with patient pain (Thompson et al., 2009) and translate as a non-traumatic way of diagnosing discogenic backpain. However, MODIC signs describe only the inflammatory state of the endplates, and not the morphology or the dynamic behavior of the disc itself. The Pfirrmann classification is another way to describe degenerative lumbar disc (Pfirrmann et al., 2001). This classification encompasses a multi-parameter description of the disc on T_2_-weighted MR images (disc height, signal intensity, etc.) allowing a grading system of the degenerative process. Several studies validate the correlation between MRI findings and histochemical composition of the intervertebral disc (Tertti et al., 1991; Benneker et al., 2005). However, none of these parameters take into account the dynamic behavior of the disc. While MRI seemed less accurate than discography to detect radial fissure (Osti and Fraser, 1992), other works suggest that successful detection is possible via MRI (Yu et al., 1988; Saifuddin et al., 1998). A specific relationship was found between radial fissure and positive discography (Aprill and Bogduk, 1992) by delineating a high intensity zone (HIZ) in the annulus fibrosus (AF), as the latter appears hyperintense compared to the nucleus pulposus (NP) on T_2_-weighted MR images. However, as HIZ is frequent among pain-free subjects, controversy exists and still persists (Khan et al., 2014).

Contrarily to qualitative T_1_- and T_2_-weighted MRI, where the signal intensity interpretation can be affected by several experimental factors, biased perception, or scanner variability, quantitative MRI provides an absolute, objective and lower-variability characterization of the imaged anatomical structures. In intervertebral discs, typically quantified parameters are the T_1_, T_2_, or T_1ρ_ relaxation times, magnetization transfer (MT) ratio, and the apparent diffusion coefficient (ADC). These parameters can potentially be used as non-invasive biomarkers for different degeneration stages since they are related to the disc histochemical composition and condition, such as for example water, proteoglycan, or collagen content, matrix integrity, loading or aging (Tertti et al., 1991; Watanabe et al., 2007; Mwale et al., 2008; Menezes-Reis et al., 2015; Hwang et al., 2016; Galley et al., 2017; Paul et al., 2018; Enokida et al., 2020). In general, mapping the relaxation time constants provides the advantage of more accurate and reproducible differentiation between the disc tissues of NP and AF and reduced segmentation variability.

To our knowledge, there is no reference available in the literature regarding MRI capability of detecting annulus fissures and characterizing their biomechanical and morphological variations as a function of bending load. We hypothesized that we could non-invasively identify an annular radial fissure and analyze the dynamic behavior of the NP migration by using optimized T_1_ and T_2_ mapping protocols to assess and quantify the degree of disc fissuring, along with the degree of nucleus displacement/deformation through the fissure, depending on the spine position.

We present here the first results of an *ex vivo* study. Quantitative and anatomical MRI scans were performed on a loaded fresh lamb spine specimen under 3 different mechanical states (neutral, flexion, extension) in both intact and damaged conditions (anterior radial fissure for the purpose of this Proof-Of-Concept study). Our first objective was to assess whether our MRI protocol allows to detect and further characterize a radial fissure within the annulus. Our second objective was to determine the deformation/displacement fields induced in the nucleus pulposus based on the imposed bending angle of flexion/extension, in both intact and damaged conditions. Our third and last objective was to elaborate a simplified finite element model from the actual geometry and boundary conditions in order to study the contribution of the radial fissure onto the stress distribution in the nucleus.

## Materials and Methods

### Specimen

We used a fresh lamb spine specimen, comprising 3 vertebrae (L1-L3) and 2 intervertebral discs (2 functional units) of the upper lumbar spine (lamb fillet). The specimen was kept at 4^*circ*^C the night before the experiment. Two functional units were kept, such that a healthy control disc could be used at all times for comparison (e.g., in case of aberrant findings).

With the exception of ligamentous and articular capsule tissue, all soft tissue was otherwise removed. In the vertebral body of the 1st and 3rd vertebra, we inserted 2 MR compatible sticks of length L = 8 cm, and diameter ∅ = 6 mm. We kept the specimen hydrated at all times by wrapping it inside saline soaked gauze (Wilke et al., 1998b).

**FIGURE 1:**
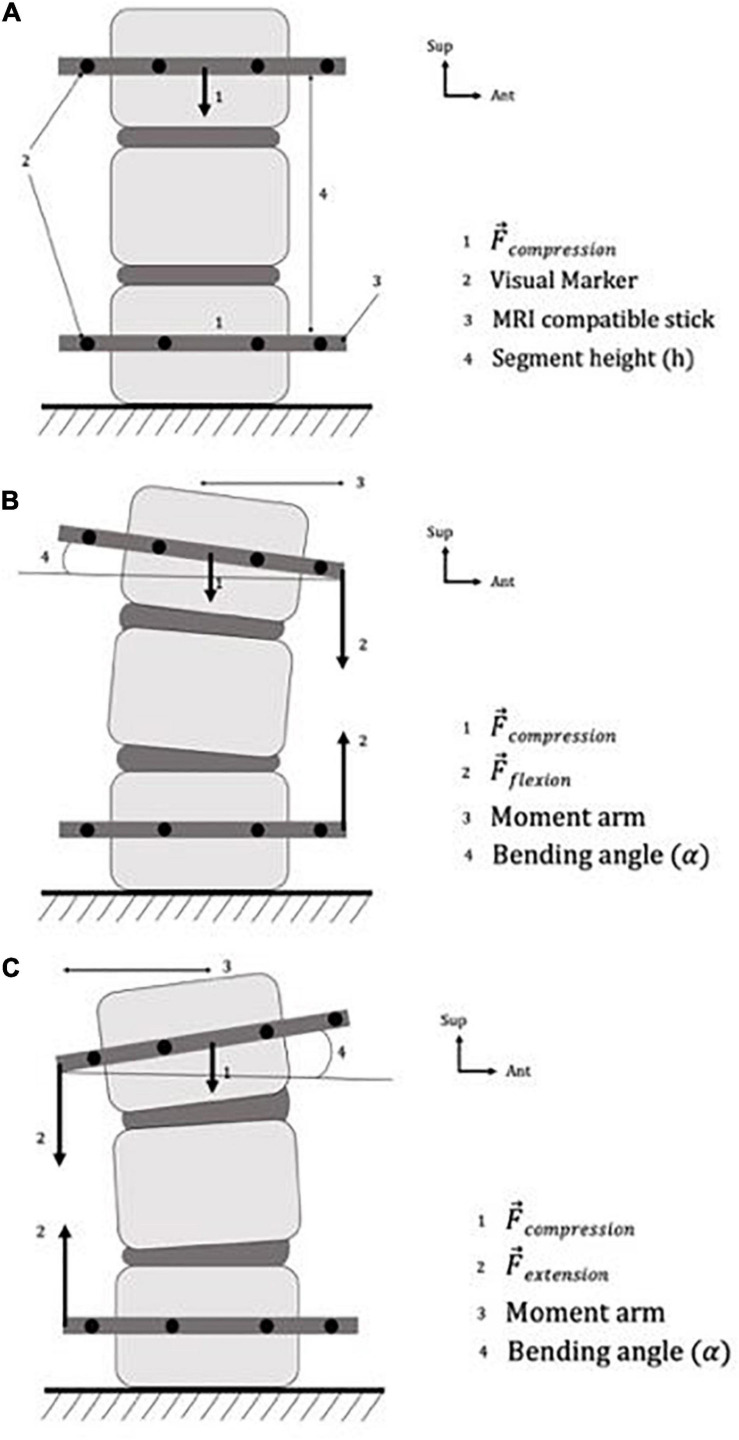
Specimen preparation and boundary conditions of the specimen, in panels **(A)** neutral position, **(B)** flexion position, **(C)** extension position.

An hour and a half prior to the experiment (Adams, 1995), to avoid over-hydration (McMillan et al., 1996), we creep-loaded the specimen using an axial compressive load of 60 N, resulting in stress of 0.03 MPa. The load was applied by an elastic compressive system, which we set on the transverse vertebral process of the 1st and 3rd vertebra. To simulate an apparent compressive load resulting from muscle activity and gravity (Callaghan et al., 1998), the compressive elastic system was maintained throughout the experiment (Figure 1A).

First, the specimen was imaged under several mechanical states with intact discs (see below). Secondly, we punctured the anterior part of the annulus using a lumbar puncture needle (18G – 1.2 mm diameter) to damage the disc. The resulting fissure represented 54% of the total height and 27% of the disc transversal diameter. We chose to damage the anterior part of the disc from its inherently easier access in comparison with the posterior part to have a perfectly reproducible lesion model. Besides, similar types of fissure have been documented using discography (Saifuddin et al., 1998) or cadaveric studies (Yu et al., 1988).

### MRI Acquisitions

All images were acquired using a 3.0-Tesla imager (Siemens Healthineers, Erlangen, Germany). Room temperature was kept constant at 24°C during the entire examination, and three different MR sequences were performed:

(1) Anatomical images were acquired using a multi-slice 2D T_2_-weighted Turbo Spin Echo (T_2_-w TSE) sequence with fat suppression, number of averages NA = 10, echo time TE = 99 ms, repetition time TR = 3800 ms, and echo train length = 21. A total of 16 axial slices with resolution 0.31 0.31 1 mm^3^, and in-plane field of view (FOV) of 84 100 mm^2^ covering the disk and part of the adjacent vertebrae were obtained in 21 min 41 s.
(2) T_2_ mapping was performed using a multi-echo Spin Echo sequence with TR = 4500 ms, and NA = 1. An echo train of 32 with first echo at 30 ms and echo spacing of 30 ms was used. A single transverse slice with in-plane resolution of 0.52 0.52 mm^2^ (FOV = 84 99 mm^2^) and slice thickness of 2 mm was acquired in 12 min.
(3) T_1_ mapping was obtained from multiple Inversion Recovery (IR) Turbo-Spin-Echo sequences with TE/TR = 14/6000 ms, NA = 1, and inversion times of 30, 60, 130, 300, 600, 1300, 3000, 5800 ms. All 8 acquisitions were performed across a single transverse slice of resolution 0.52 0.52 mm^2^, with corresponding FOV = 84 99 mm^2^ and slice thickness of 2 mm, acquired in 18 min.

We used the same imaging protocol for each mechanical state of the specimen, resulting in a total of 18 scans. Axial slice orientation was chosen to provide a better visualization and a more appropriate modeling of the NP, the AF and its fissure within the disc with respect to the sagittal view.

### Image Processing

Images were processed using in-house scripts and functions developed with MATLAB (MathWorks, Natick, MA, United States). T_1_ and T_2_ maps were obtained after normalizing the data and fitting them pixel-wise using either an exponential build-up (*S*_1_) or decay (*S*_2_) model:

$$S_{1}=a\cdot \left(1-2\cdot e^{t/T_{1}}\right)$$

$$S_{2}=b\cdot e^{-t/T_{2}}$$

With *S*_*i*_ the normalized signal intensity in arbitrary units, *t* the time obtained from the echo train or inversion times, and *a/b* constants of the fitting models. Relaxation times are tied to the magnetic properties of the different tissue species and are expected to vary significantly between the different disc compartments.

### Mechanical Loading

We controlled the boundary conditions by fixing the lower vertebra and using rubber bands in different configurations depending on the various elastic systems targeted. The first rubber band applied axial compressive loading (as described above), and the second one applied bending loading (either in extension or in flexion). We used this elastic loading system to modify the position of the specimen in the sagittal plane: flexion was created by applying a bending load on the anterior part of the sticks (Figure 1B) and extension on the posterior part (Figure 1C). Our loading protocol is described in Table 1.

**TABLE 1:** Mechanical states and the corresponding estimated boundary conditions of the specimen.

**Step**	**Mechanical loading state**	**Axial compression (N)**	**Bending force (N)**	**Bending moment (N.m)**
0	Neutral	0	0	0
1	Neutral (Figure 1A)	59.7	0	0
2	Flexion (Figure 1B)	59.7	81.1	2.1
3	Extension (Figure 1C)	59.7	80.9	2.8
**Fissure**				
4	Extension (Figure 1C)	59.7	80.5	2.8
5	Flexion (Figure 1B)	59.7	81.0	2.1
6	Neutral (Figure 1A)	59.7	0	0

### Measurements

We used a mark tracking system to calculate the bending angle and the axial displacement (Figure 1A). In particular, we placed visible marks on the sticks and on the vertebra specimens, and measured their respective positions relative to each other before and during each loading step (Germaneau et al., 2016).

We calculated the bending angle using the following process:

• We determined the baseline angle made by the upper MRI-compatible sticks and the horizontal one (α_0_) from the specimen in neutral loading.
• The same measurement was repeated with the bending loading, i.e., flexion or extension (α).
• We subtracted α_0_ to α and obtained the corresponding bending angle. A negative value represents a flexion, and a positive value an extension.

We calculated the axial strain from the following process:

• In resting state, we determined the overall height of the vertebral specimen by measuring the distance between the two upper parts of the MRI-compatible sticks (h_0_).
• The same measurement was repeated with mechanical loading (h).
• We computed the axial strain as the variation of h relative to the baseline value h_0_ at resting state. A negative value represents a height reduction (i.e., a compression) and positive value a height increase.

We compared bending angles and strain values after each loading step. We evaluated the measurement uncertainty (from measurement repetition on known imposed values) at 0.1% for axial strain and 0.1° for angle variation (Germaneau et al., 2016).

**FIGURE 2:**
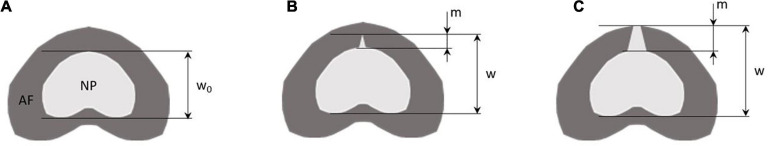
Method to calculate the nucleus sagittal boundary displacement (w), the degree of nucleus pulposus (NP) migration (m) within the annulus fibrosus (AF), and the nucleus sagittal strain for each mechanical step. Panel **(A)** is the reference mechanical loading state, i.e., step 1, panel **(B)** is a mechanical state with partial migration of the annulus m < 50% of the total annulus thickness, panel **(C)** is a mechanical state at total migration of the annulus m > 50% of the total annulus thickness. Nucleus sagittal boundary displacement is calculated for each mechanical state by w – w_0_. Nucleus sagittal strain is calculated for each mechanical state by (w – w_0_)/w_0_. Migration m is calculated from the proportion of nucleus displacement within the annulus.

Furthermore, we measured nucleus displacement and inferred its strain in the sagittal direction from the T_1_ maps. Displacement on sagittal disk boundaries was calculated as the relative difference in distance between the nucleus boundaries at the reference mechanical state (step 1) and the studied state (e.g., step 3, Figure 2).

**FIGURE 3:**
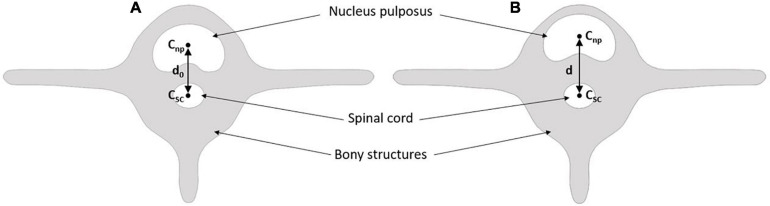
Method to calculate global nucleus sagittal displacement. Panel **(A)** is the reference mechanical loading state, i.e., step 1. Panel **(B)** is the studied mechanical state, i.e., step 2 to 6 (Table 1). C_*np*_ is the centroid of the nucleus pulpous; C_*sc*_ is the centroid of the spinal cord; d_0_ is the reference distance; d is the distance of the studied mechanical state.

Total sagittal nucleus displacement was computed as the relative difference between the distances of the nucleus and spinal cord centroids at the reference state (step 1) and each studied step (e.g., step 4). To determine the position of each centroid, we segmented and isolated the nucleus pulposus and the spinal canal from the T_1_ maps (Figure 3). The spinal cord is a fixed and identifiable structure that provides a good reference for the centroid tracking process. Nucleus displacement was obtained with an uncertainty of 0.05 mm.

We inferred the nucleus sagittal strain according to:

ε=(w-w0)/w0

where w0 is the distance between anterior and posterior nucleus boundaries at step 1 (Figure 2A, reference state), and w is the distance between anterior and posterior nucleus boundaries at the investigated steps (Figures 2B,C).

We further evaluated the degree of migration of the nucleus in the fissure during the different mechanical loading steps. This migration was calculated as the ratio between the displacement of the nucleus in the fissure, and the annulus thickness (Figure 2).

### Finite Element Model

**FIGURE 4:**
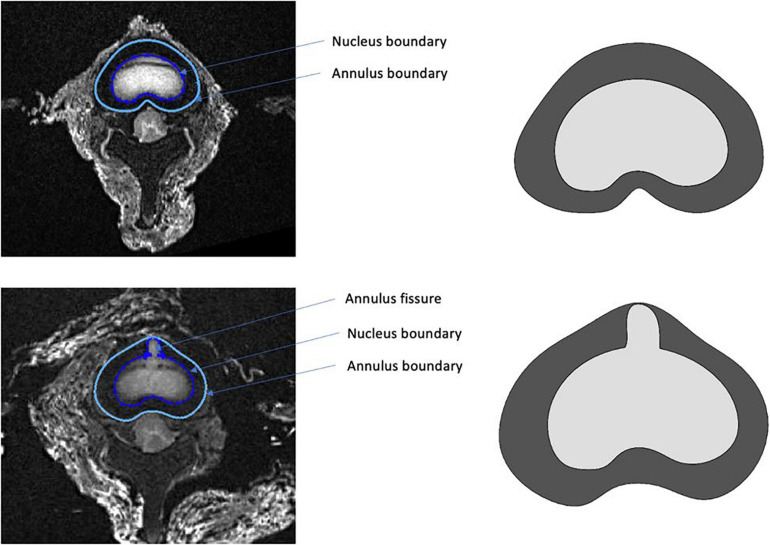
Example of segmentation performed in the intact (top) and fissured (bottom) states, used as input for the finite element model.

A finite element (FE) model was developed to perform a stress analysis from a simplified geometry extracted using MRI. Our aim was to use this specific FE model from the actual geometry and the actual boundary conditions in order to calculate the stress distribution in the nucleus and the contribution of the radial fissure. FE analysis was carried out using SolidWorks software (Dassault Systèmes Corporation, Waltham, MA, United States). The geometry of the model was defined using points extracted from the boundary of the annulus and nucleus segmented based on a threshold on the MR images acquired in the intact and fissured states (Figure 4). For this stress analysis, homogeneous behavior was considered to model materials (Shin et al., 2007), of which the properties are specified in Table 2.

**TABLE 2:** Young Modulus and Poisson ratio used for the finite element model of the disc.

	**Young modulus (MPa)**	**Poisson ratio**	**Reference**
Nucleus	1	0.499	Shin et al., 2007
Annulus	8.4	0.45	

The boundary conditions applied to the endplates of the FE model corresponded to the loading imposed during experiments to create bending moments. For that operation, moment was applied on the upper plate of the disk and displacement was considered null on the inferior plate. From there, axial stress and intensity of shear stress induced in the nucleus could be obtained.

## Results

### MR Imaging Results

**FIGURE 5:**
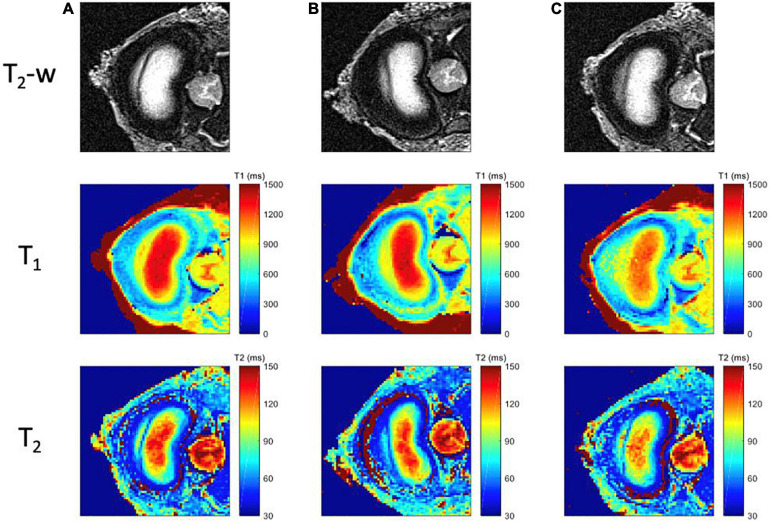
MR images for each mechanical loading state of the intact specimen. First row corresponds to the T_2_-weighted images; second row to the computed T_1_ maps, with a scale ranging from 0 ms (blue) to 1500 ms (red); and third row to the T_2_ maps, with a scale ranging from 20 ms (blue) to 150 ms (red); for three different positions **(A)** Neutral (step 1) **(B)** Flexion (step 2), **(C)** Extension (step 3).

**FIGURE 6:**
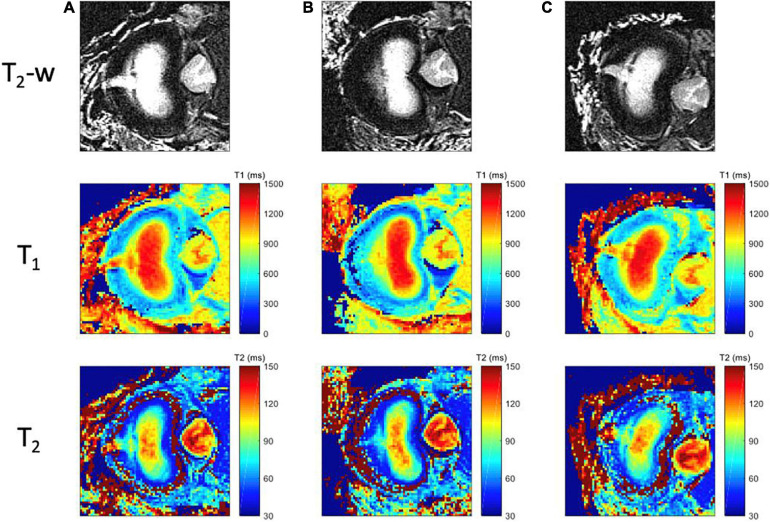
MR images for each mechanical loading state of the specimen with radial tear. First row corresponds to the T_2_-weighted images; the second row to T_1_ maps, with a scale ranging from 0 ms (blue) to 1500 ms (red); and third row to the T_2_ maps, with a scale ranging from 20 ms (blue) to 150 ms (red); for three different positions **(A)** Neutral (step 6), **(B)** Flexion (step 5), **(C)** Extension (step 4).

Examples of T_2_-weighted images, T_1_ and T_2_ maps are presented in Figures 5, 6 for each mechanical state of the specimen, with or without radial fissure radial fissure, respectively. On Figure 5, the anterior fissure is clearly visible for each MRI sequence.

According to the Dallas classification (Sachs et al., 1987), the analyses of the T_2__–_w TSE, T_1_ and T_2_ maps reveals a fissure shape similar to a grade II discogram in neutral and extension positions, whereas it is similar to a grade I discogram in flexion position. T_1_ and T_2_ maps reveal tissue infiltration within the fissure in neutral and in extension positions based on the respective relaxation times measured in the different compartments. This infiltration is no longer visible in flexion position of the specimen, and the magnetic properties of the infiltrated tissue are similar to those of the annulus fibrosus (T_1_ > 800 ms, T_2_ > 80 ms). These maps confirm that the nucleus deformed in accordance with the loading direction in a greater extent when the annulus is fissured.

### Total Disc and Nucleus Mechanical Displacements and Strains

Axial strain, bending angle variation, nucleus boundary sagittal displacement, nucleus total sagittal displacement, and nucleus sagittal strain for each mechanical condition are summarized in Table 3. Steps 1 to 3 (Table 1) present our findings for the intact specimen at different mechanical states. Between steps 3 and 4, an experimental anterior fissure was performed at the annulus fibrosus site. Accordingly, steps 4 to 6 present the results for the damaged specimen.

**TABLE 3:** Axial strain, angle variation, nucleus boundary sagittal displacement, nucleus global sagittal displacement, nucleus sagittal strain, migration of the nucleus in the annulus and cumulative time under axial compression for each mechanical loading step.

**Step**	**Axial strain^1^**	**Specimen angle^2^**	**Angle variation^3^**	**Nucleus boundary sagittal displacement^4^**	**Nucleus global sagittal displacement^4^**	**Nucleus sagittal strain^1^**	**Migration of nucleus in the annulus^5^**	**Cumulative time under axial compression**
1 (Neutral)	−4	4.7	–	–	–	–	–	lh30
2 (Flexion)	−7.3	−8.8	−13.5	−2.2	−3.6	−5.6	0	3h
3 (Extension)	−8.3	10.4	5.7	2.2	−0.9	−6.1	0	4h30
**Fissure**								
4 (Extension)	−8.8	11.1	6.4	5.0	3.7	21.1	100	6h30
5 (Flexion)	−7.5	−8.9	−13.6	−0.6	−1.6	−3.1	21	8h30
6 (Neutral)	−4.6	8.7	4.0	4.5	1.5	20.8	100	10h

*^1^In percentage, negative value corresponds to a decrease of the height of the specimen. ^2^In degrees, negative value corresponds to a flexion angle, positive value to an extension one. ^3^In degrees, negative value corresponds to a flexion angle, positive value to an extension one. This angle corresponds to the actual specimen angle respective to the baseline’s one (step 1 neutral). ^4^In millimeters, negative value corresponds to a posterior displacement, positive value to an anterior one. ^5^In percentage.*

Axial strain results show an increase with time when comparing two steps with the same mechanical state, e.g., steps 1 and 6. Specimen angle results also show an increase with time. There is a considerable change in nucleus behavior before and after fissuring the annulus. Displacement of the nuclear boundaries increases with extension after the fissuring (+2.8 mm), whereas posterior displacement decreases with flexion (−1.6 mm). The same behavior is visible for nucleus global displacement (+4.6 mm for extension and −2 mm for flexion). The strongest effect of the fissure is observed on the sagittal strain (+27% in extension and +2.5% in flexion). Both flexion and extension with an intact nucleus lead to nucleus compression (negative strain values of −5.6% and −6.1% respectively). In contrast, with a fissured annulus, only flexion leads to nucleus compression (−3.1%), whereas extension causes its stretching (+21.1%).

### Stress Analysis

**FIGURE 7:**
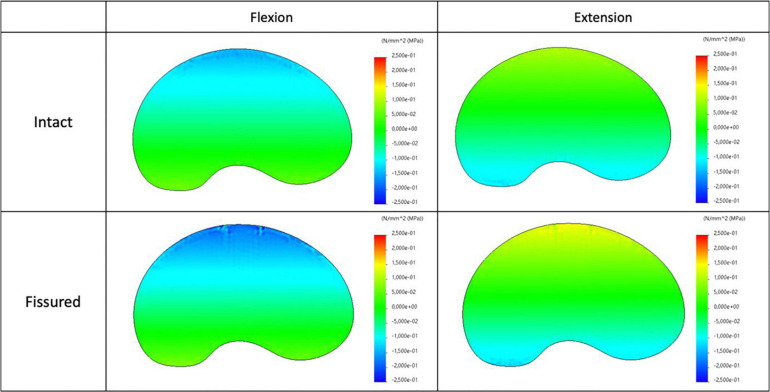
Distribution of the axial stress map for intact (top) and fissured (bottom) disc with flexion (left) and extension (right) loading.

**FIGURE 8:**
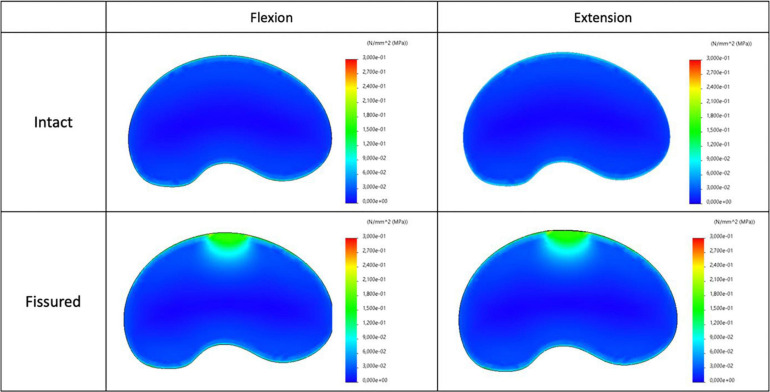
Distribution of the intensity of maximal shear stress for intact (top) and fissured (bottom) disc with flexion (left) and extension (right) loading.

The maps of axial stress and intensity of maximal shear stress for intact and fissured specimen are shown in Figures 7, 8, for both flexion and extension loading. For the intact specimen, we observed classical behavior with compressive and tensile components in accordance with bending loads. For the fissured specimen, we observed increased stress value in the vicinity of the fissure (around 0.2 MPa). The distribution of the intensity of maximal shear stress further exacerbates this difference by displaying increased values in front of the fissure (around 0.2 MPa), whereas maps were homogeneous in the intact disc.

## Discussion

### Original Findings

Our first and secondary aims were to detect radial fissure of the annulus fibrosus using quantitative MRI, and to characterize the biomechanical behavior of the fissured and intact intervertebral disc, respectively. We defined an original protocol combining qualitative and axial quantitative MRI, computed segmentation, mark tracking analysis and FE modeling. This original approach enabled characterizing the morphology and the biomechanics of the radial fissure the nucleus pulposus according to sagittal bending load and the presence / absence of an anterior radial fissure.

By comparison, classical MRI approach assess either the grade of degeneration (Pfirrmann et al., 2001), the inflammatory status of the endplate (Modic et al., 1984), the presence of a high intensity zone in the annulus (Aprill and Bogduk, 1992) or the external geometry of the disc (Fardon et al., 2014). None of these parameters are a direct observation of a radial fissure. In addition, discography allow a morphologic characterization of the fissure but it is invasive (Carragee et al., 2009) and does not explore the dynamic nature of this fissure (Bogduk, 2013).

Data from this proof-of-concept study tend to confirm the dynamic nature of the nucleus pulposus under the influence of bending loads, i.e., deformation/displacement of the nucleus pulposus away from the direction of the load. A systematic review confirmed this behavior for healthy discs, but findings were conflicting regarding pathological discs (Kolber and Hanney, 2009). We can highlight that none of the papers included in this review used a protocol similar to ours.

Our specimen was prepared according to the guidelines for spinal cadaveric studies by controlling for hydration and axial creep load (Adams, 1995; Wilke et al., 1998b). Due to experimental limitations, the axial compressive load was lower than the recommended value (60 N versus 300 N in the guidelines) and was instead applied longer in order to reach the equivalent effect (90 min versus 30 min in the guidelines).

Adjunction of quantitative imaging improves the precision of disc morphometric characterization and enables experimenters to measure both nucleus displacement with good accuracy (0.1 voxel) and strain under various loading conditions (flexion-extension). By comparing the T_1_ relaxation times of the tissues at the fissure level to those of the nucleus, it appears reasonable to claim that the nucleus moves toward/deformed into the fissure under bending load influence. Our study showed that T_1_ and T_2_ values are relevant parameters for automated segmentation of the different disc regions. This justifies future effort in developing fast quantitative acquisitions that will offer objective metrics for the analysis of the disc biomechanics. Indeed, and as indicated by the plethora of studies in the field, there is a clear need of establishing robust quality criteria for image analyses used in clinical research and clinical trials. This task is all the more difficult as conventional MR images display only shades of gray that are very dependent on the hardware (coil profile, field strength, field homogeneity) and the operator (sequence parameters, patient positioning, signal intensity thresholds for segmentation, etc.).

When comparing two steps within the same mechanical state, e.g., steps 2 and 5, an increase of axial strain and bending angle with time was expected. This corresponds to the creep load induced by compressive axial load. However, between step 1 (intact disc in neutral state) and step 6 (fissured disc in neutral state) the angle difference is + 4°, indicating that the specimen’s position shifted from neutral toward extension even though the elastic bending system had been removed. This value is much greater than what we observed for the other two mechanical states (+ 0.7° for extension and − 0.1° for flexion). This observation, albeit not expected, could explain why MRI and mechanical results of step 6 (fissured disc in neutral state) are very close to those of step 4 (fissured disc in extension). This sizable increase of extension angle in step 6 could be a consequence of the manually performed anterior fissure and should be further explored in future studies.

Our results also revealed a link between disc abnormality and range of motion by using a non-invasive diagnostic method differing from interventional intra-discal procedures. While discography highlights the physical presence of a fissure within the annulus under fixed positional conditions, it remains impossible to observe the migration of the nucleus under postural influence (Walker et al., 2008). Using our non-invasive MRI approach, we observed and quantified the migration of the nucleus into the annulus from the quantitative maps, under conditions equivalent to the physiological loading (up to 5-6 degrees per level) usually observed for daily range of motion in humans (Alini et al., 2008). In this work, the artificial fissure induced considerable damage including the migration of the nucleus within the full thickness of the annulus (100% of migration). As a result, the nucleus strain increased with stretching in the sagittal direction inducing reduction of internal pressure. Yet, flexion partially restored not only the nucleus morphology with observed migration dropping from 100 to 21%, but also the internal pressure with a measured compression strain of 3.1% restoring internal pressure, and consequently physiological capabilities of the disc. These observations confirm the dynamic behavior changes of the fissure.

Ultimately, we used a simplified finite element model, based on geometry directly extracted from MRI data. This approach was enriched by the real boundary conditions quantified during the experiments. This dual approach enabled simultaneous measurement of stress and strain fields on the nucleus, thereby providing a complete biomechanical analysis of the normal and pathological disc. From a clinical point of view, the migration of the nucleus pulposus alongside the fissure could become a nociceptive trigger, leading patient pain. Furthermore, in this case, the singularity and its evolution observed from MRI maps can be linked to stress variation in the nucleus with an increasing of shear components.

### Study Limitations

Despite being encouraging, our results suffer from several limitations. This study is a proof-of-concept study performed on only one disc. Repeating the protocol on multiple samples would allow us to assess the reproducibility of our method and to draw more robust conclusions from a statistical point of view. Another limitation could arise from the animal nature of our specimen and its difference with human discs. Indeed, quadrupedal station induces geometrical changes (O’Connell et al., 2007), variations in mechanical properties (Alini et al., 2008) and chemical composition of the disc (Zhang et al., 2014). Nonetheless, a recent review (Daly et al., 2016) concluded that ovine disc is a reasonable choice for preliminary biomechanical or injury model studies. Animal models are currently used in surgical, biomechanical (Casaroli et al., 2017) and histological studies (Schollum et al., 2008). Rather than species type, age and degeneration state of the specimen might actually have a stronger impact on the results since both of these parameters alter disc biomechanics (Adams et al., 1996). With age and degeneration, there is a decrease in the disc notochordal cell population. This triggers a reduction of proteoglycan secretion, leading in turn to a drop in nuclear hydration. Type II collagen is progressively replaced by type I collagen, leading to a more fibrous nucleus (Adams and Roughley, 2006). All of these processes impact the mechanical behavior of the nucleus according to motion and can affect the magnetic properties, such as T_1_ and T_2_. As our specimen comes from a young animal with no sign of degenerative disc, the results could not be generalized beyond these criteria. Another limitation comes from the type of fissure induced in our study. Our experimental setup ensured good access to the anterior part of the disc without damaging the specimen. Though rare, anterior fissures are encountered in discography (Saifuddin et al., 1998) or cadaveric studies (Yu et al., 1988). However, most of the fissures associated with discogenic backpain are either posterior or postero-lateral (Bogduk et al., 2013). Because of the different shape and thickness of the anterior annulus (Cassidy et al., 1989), results could be different with a posterior fissure. Ultimately, MRI examination time needs to be shortened to envision *in vivo* studies. Since ours was the first study of its kind, basic MR sequences were used in order to assess the feasibility of T_1_ and T_2_ maps as markers to investigate the disc biomechanics. As a consequence, repeating the current imaging protocol in the 3 different conditions leads to a 6-h examination. Such scan time did not have an impact on our specimen since it has been shown that no change in mechanical properties is observed after 20 h of testing (Wilke et al., 1998a), but faster acquisition schemes need to be implemented to allow a potential transfer to patients *in vivo*. We propose to implement strategies that enable simultaneous acquisitions of T_1_ and T_2_ (Ma et al., 2013) in future studies.

Concerning FE analysis, we used a simplified model to determine stress distribution according to imposed motion and the presence of a fracture in the annulus. Even if we used real geometry and boundary conditions read during experiments, our model had some limitations. As already performed for previous works from literature, we considered linear behaviors and isotropic properties (Shin et al., 2007; Zanjani-Pour et al., 2016). This approach was sufficient in the present work to analyze stress distribution. However, to validate the model, it would be necessary to identify the mechanical properties of tissues and to implement them in the model which could be validated from comparison of displacement and strain fields. Furthermore, we did not model the facet joints in accordance with the limited values of imposed moments (3 N.m). According to our experiments, facet joints were not activated for this magnitude of applied loads, however, they would have to be modeled for higher imposed moments.

Future directions would include replicating this study using a greater number of ovine discs, along with inducing different orientations for the radial fissure in order to better reflect clinical conditions, and applying additional movement directions, typically including the coronal and frontal plans. If results are consistent, our ambition would be to design the next phase of *ex vivo* studies using human cadaveric discs at different levels of degeneration and age, and thereby make it possible to transfer our MRI methods to *in vivo* clinical applications.

### Future Clinical Implications

Once adapted to *in vivo* experimentations and combined with classical assessment tools (MRI, X-ray, etc.), such protocol would help clinicians to assess patients with discogenic pain and/or radicular pain due to disc herniation. Together with the directional preference concept (May and Aina, 2012; May et al., 2018), it would help to identify patients who could benefit from a physiotherapeutic mechanical treatment (McKenzie and May, 2003) from those who need surgery. We believe that this protocol would ease the clinical decision-making process, and hence optimize patient care as well as reduce health cost related to back pain.

## Conclusion

In this Proof-of-Concept study, we demonstrated the possibility to characterize the morphological and biomechanical parameters of a radial fissure within an *ex vivo* ovine disc. To do so, we combined quantitative T_1_ an T_2_ mapping MRI, T_2_-weighted MRI, computed segmentation, mark tracking analysis and finite element modeling.

Each MRI sequence allowed a clear and original visualization of the discal damage, i.e., radial fissure, non-invasively. The nucleus pulposus moved anteriorly with extension bending load and posteriorly with flexion bending load. After the anterior annulus was damaged, the nuclear displacement and strain increased for extension load and decreased for flexion load. The displacement and strain of the nucleus appeared to follow the direction of the fissure and the direction of the bending load.

This preliminary work, once validated on a larger scale, could have substantial applications for:

• radio-clinical non-invasive disc explorations and correlations with patient lumbar pain,
• dynamic characterization of the disc under physiological and pathological conditions.

## Data Availability Statement

The raw data supporting the conclusions of this article will be made available by the authors, without undue reservation.

## Author Contributions

J-PD, TV, and PR: conceptualization. AG, MY, MS, and NS: methodology. J-PD, AG, MY, MS, and NS: software and validation. MS and NS: resources. J-PD, AG, NS, and PR: writing—original draft preparation. J-PD, TV, AG, MB, MR, NS, and PR: writing—review and editing. AG, NS, and PR: supervision and project administration. NS, MS, and PR: funding acquisition. All authors have read and agreed to the published version of the manuscript.

## Conflict of Interest

The authors declare that the research was conducted in the absence of any commercial or financial relationships that could be construed as a potential conflict of interest.
